# Expression of a Grape *VqSTS36*-Increased Resistance to Powdery Mildew and Osmotic Stress in Arabidopsis but Enhanced Susceptibility to *Botrytis cinerea* in Arabidopsis and Tomato

**DOI:** 10.3390/ijms19102985

**Published:** 2018-09-30

**Authors:** Li Huang, Xiangjing Yin, Xiaomeng Sun, Jinhua Yang, Mohammad Zillur Rahman, Zhiping Chen, Xiping Wang

**Affiliations:** 1 State Key Laboratory of Crop Stress Biology in Arid Areas, College of Horticulture, Northwest A&F University, Yangling 712100, China; huanglimakeit@163.com (L.H.); yinxiangjingsmile@163.com (X.Y.); sxm1593843858@163.com (X.S.); 13759927085@163.com (J.Y.); zillurhrc_1976@yahoo.com (M.Z.R.); 2 Key Laboratory of Horticultural Plant Biology and Germplasm Innovation in Northwest China, Ministry of Agriculture, Northwest A&F University, Yangling 712100, China; 3 Shanghai Vocational College of Agriculture and Forestry, Shanghai 201699, China; chenzp@shafc.edu.cn

**Keywords:** stilbene synthase, grape, powdery mildew, *Botrytis cinerea*, salt stress, drought stress, tomato, arabidopsis

## Abstract

Stilbene synthase genes make a contribution to improving the tolerances of biotic and abiotic stress in plants. However, the mechanisms mediated by these *STS* genes remain unclear. To provide insight into the role of *STS* genes defense against biotic and abiotic stress, we overexpressed *VqSTS36* in *Arabidopsis thaliana* and tomato (Micro-Tom) via *Agrobacterium*-mediated transformation. *VqSTS36*-transformed Arabidopsis lines displayed an increased resistance to powdery mildew, but both *VqSTS36*-transformed Arabidopsis and tomato lines showed the increased susceptibility to *Botrytis cinerea*. Besides, transgenic Arabidopsis lines were found to confer tolerance to salt and drought stress in seed and seedlings. When transgenic plants were treated with a different stress, qPCR assays of defense-related genes in transgenic Arabidopsis and tomato suggested that *VqSTS36* played a specific role in different phytohormone-related pathways, including salicylic acid, jasmonic acid, and abscisic acid signaling pathways. All of these results provided a better understanding of the mechanism behind the role of *VqSTS36* in biotic and abiotic stress.

## 1. Introduction

Stilbene synthase (*STS*) is a key enzyme for the synthesis of phytoalexin stilbenoids through the polyphenol biosynthetic pathway [1]. These stilbenoids can be produced by a limited number of plants species, including red pine, peanut, sorghum, and grape [2,3,4,5]; they are secondary metabolites of plants with capacities of resistance against different environmental attacks [6,7], such as infection by pathogens, salinity stress, or drought stress. However, the potential mechanism of *STS* genes in pathogens resistance and its possible differential responses are still unclear. Further exploration is required to investigate precise relationship between *STS* genes and biotic or abiotic stress.

Grapevine is an economically important crop, but it is easily subjected to various pathogen diseases [8]. Major fungal diseases, including powdery mildew, *Botrytis cinerea*, and downy mildew, can cause severe yield losses and decline in fruit quality. It has been demonstrated that *Vitis* species, such as *Vitis vinifera*, *V. labrusca*, and *V. rupestris*, showed various levels of resistance to powdery mildew disease caused by the fungus *Uncinula necator* [9]. Therefore, to reduce the threat of powdery mildew disease on grape, it is feasible to use genetic materials from wild grape species to develop resistant cultivars. Powdery mildew is a biotrophic pathogen, while the fungus *B. cinerea* is a necrotrophic pathogen which colonizes dead plant tissues and leads to gray mold in fruits. These fungi release a set of effector proteins into host cells to intervene the host immunity [10], including activating a hypersensitive response and changing the expression profile of defense-related genes [10,11]. Invasion by pathogens can induce relative plant hormones to respond. Generally, salicylic acid (SA) is effective against biotrophic pathogens, whereas Jasmonic acid (JA) is active against necrotrophic fungi [12]. Expression of *STS* genes can also be increased in response to plant hormones, such as SA and jasmonate acid (JA) [13,14,15]. Exogenous SA can reduce the cytosine DNA methylation of certain *STS* genes, which results in significant increase in the expression of *STS* genes and the accumulation of resveratrol in the cell cultures of *V. amurensis* [16].

Water deficit is also a major limiting factor for economic agricultural products [17,18], crop yield [19,20]. Furthermore, due to global warming, there will be an increase in aridity [17,21], which will aggravate the salinization of land in some areas; Therefore, a water deficit becomes another important limiting factor for grape yield and quality [17,22]. Osmotic stresses, including drought and salinity stresses, not only have negative impacts on root development [23] but also cause an accumulation of osmotic compounds and ions, which activate detoxifying processes [24,25], thus threatening grapevine growth and development. In previous investigations, most research focused on the influence of pathogen challenges or UV radiation stress on the changes of *STS* gene expression and the accumulation of stilbene compounds; however, the role of *STS* genes in osmotic stress is still unknown. Besides, some of *STS* genes also contributed to an increase tolerance of salt and drought stresses [6,26], as well as disease resistance in grape. Therefore, functional *STS* genes with biotic and abiotic stresses can be exploited for new grape breeding resources in the future.

In the current study, we selected *VqSTS36* gene based on our previous research. We inoculated Chinese wild *Vitis quinquangularis* with powdery mildew and assessed the expression profiles of 31 *STS* genes. *VqSTS36* displayed a response to this pathogen. Specifically, the expression levels of *VqSTS36* increased until reaching a peak at 12 h post-inoculation, which was earlier than other genes, and then declined at subsequent time points [26]. As such, we selected the *VqSTS36* gene for all further functional analyses in this study. We transformed *VqSTS36* in Arabidopsis and tomato to investigate what role *VqSTS36* plays in defense against different pathogen diseases and to allow understanding of how *VqSTS36* works in the resistance to osmotic stress.

## 2. Results

### 2.1. Transgenic VqSTS36 Arabidopsis Increase Resistance against Powdery Mildew

**Figure 1:**
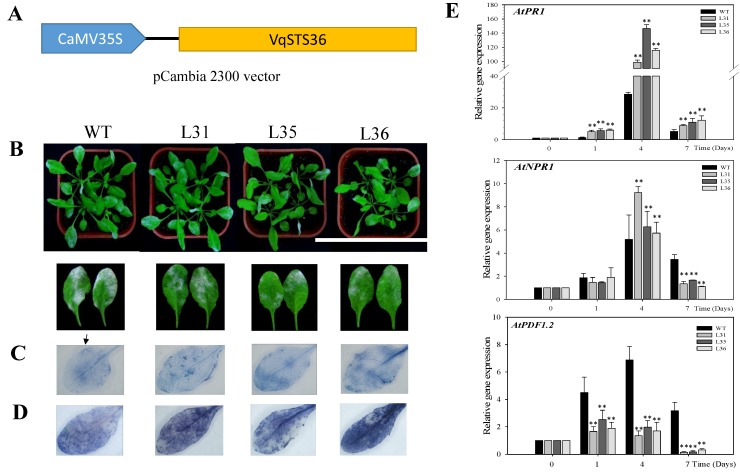
Performance of *VqSTS36* transgenic Arabidopsis following inoculation with powdery mildew and expression of defense-related genes. (**A**) Vector map of pCambia 2300:CaMV35S-*VqSTS36*. (**B**) Representative images of plants were taken at 7 days post-inoculation (dpi). (**C**) Trypan blue to detect cell death on leaves was used at 4 dpi. (**D**) Nitro blue tetrazolium (NBT) staining to detect superoxide anion accumulation, as noted by arrows. (**E**) Relative expression levels of defense-related genes in leaves collected at 0, 1, 4, and 7 dpi. Data represent mean values ± SD (with values from time point 0 hours post-inoculated (hpi) set to 1) from three independent experiments. Asterisks indicate significant differences between wild-type (WT) and transgenic lines as determined by Student’s *t*-test. (** *p* < 0.01).

Transformed Arabidopsis lines (L31, L35 and L36) and wild-type (WT) were inoculated with powdery mildew. Observation of leaf surfaces of transgenic lines displayed that the lesion coverage area was smaller than WT at 7 days post-inoculation (dpi), see Figure 1B, thus *VqSTS36* enhanced the disease resistance of transgenic Arabidopsis. To figure out the potential pathways causing the difference between *VqSTS36* transgenic Arabidopsis and WT in response to powdery mildew, we measured the cell death and superoxide anions (O_2_^−^) in inoculated leaves. As proven by the increased staining in inoculated leaves after being stained with trypan blue, see Figure 1C, and nitro blue tetrazolium (NBT), see Figure 1D, transgenic lines accumulated a greater number of cell death and more O_2_^−^ than WT. Analysis of qPCR assays for relative gene expression of SA- and JA-related genes, see Figure 1E, in transformed lines and WT at 0, 1, 3, 4 and 7 dpi, respectively. *AtPR1* and *AtNPR1* are the key components in the SA-mediated signal pathway. Gene expression of *AtPR1* reached a peak at 4 dpi, and was significantly higher than WT. Meanwhile, *AtNPR1* expression reached a peak value of 4 dpi and showed the level of expression was higher than WT. Conversely, *AtPDF1.2*, a very important gene in the JA biosynthetic pathway, was increased in both transgenic Arabidopsis lines and WT, but *AtPDF1.2* expression in transgenic plants were much lower than in WT after a value of 1 dpi following the infection of powdery mildew, see Figure 1E.

### 2.2. Over-Expression of VqSTS36-Enhanced Arabidopsis and Tomato Susceptibility to Botrytis Cinerea by Inducing the SA-Mediated Signaling Pathway

Powdery mildew is a biotrophic pathogen, *VqSTS36* increases the resistance against it in Arabidopsis by activating an SA signal pathway. However, in this case, to investigate what kind of signaling metabolic pathway would be involved when *VqSTS36* transgenic plants were subjected with the necrotrophic pathogen, *B. cinerea*, we assessed the influence of *B. cinereal* infection on plants.

**Figure 2:**
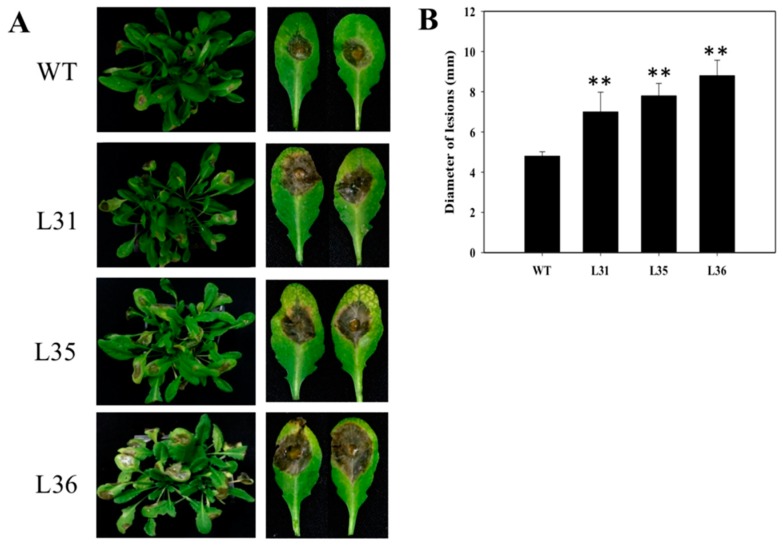
The performance of VqSTS36 transgenic Arabidopsis subjected with *Botrytis cinerea*. (**A**) The symptom of plants subjected with *B. cinerea* after 5 days. (**B**) Statistics of lesions diameter. Data represent mean values ± SD from three independent experiments. Asterisks indicate significant differences between WT and transgenic lines as determined by Student’s *t*-test (** *p* < 0.01).

**Figure 3:**
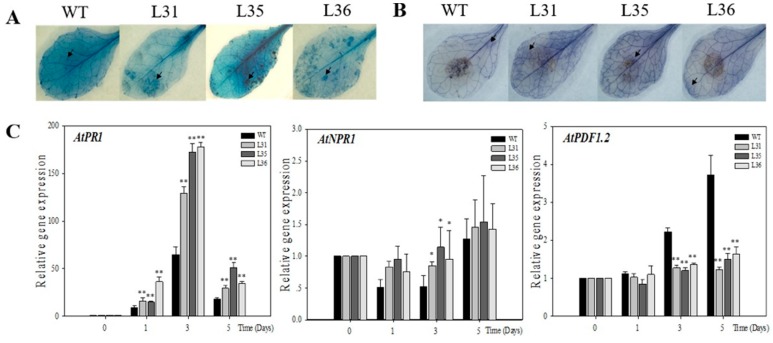
Expression of defense-related genes after VqSTS36 transgenic Arabidopsis inoculated with *Botrytis cinerea*. (**A**) Trypan blue staining to detect cell death after 3 days plants subjected with *B. cinerea.* (**B**) Trypan blue staining to detect cell death. (**C**) Relative expression levels of defense-related genes in leaves collected at 0, 1, 3 and 5 days post-inoculation (dpi). Data represent mean values ± SD (with values from time point 0 hpi set to 1) from three independent experiments. Asterisks indicate significant differences between WT and transgenic lines as determined by Student’s *t*-test (* 0.01 < *p* < 0.05; ** *p* < 0.01).

First, after infected transgenic and WT Arabidopsis with *B. cinerea* for 5 days, the leaves presented chlorosis, oil-like lesions, and visible mycelium. However, transgenic plants showed more the number of diseased leaves than WT, and the lesion diameters of the transgenic lines (0.7 to 0.85 cm) were larger than WT (0.48 cm, Figure 2A,B), which suggested the degree of decaying leaves from transgenic plants was more serious than that of WT. Three days after inoculation, the accumulations of dead cells and O_2_^−^ in the leaves were detected by trypan blue, see Figure 3A, and NBT staining, see Figure 3B, respectively. It was found that both these two indexes accumulated in transgenic Arabidopsis leaves were more than that in WT. Real-time PCR was used to analyze the expression levels of resistance-related genes in *A. thaliana* after inoculation. The results exhibited that *AtPR1* gene, a key gene in SA signaling pathways, was up-regulated in both transformed Arabidopsis and WT from 1 dpi onward. But compared to WT, relative expression of *AtPR1* were much higher in transgenic Arabidopsis, especially at 3 dpi, with values reached a peak (129 to 178) that was two to three times as high as that of WT Arabidopsis; however the relative expression of *AtNPR1* gene in transgenic lines and WT were slightly up-regulated at 5 dpi, and there was almost no significant different, see Figure 3C. The relative expression of *AtPDF1.2*, a JA-related signal-responsive gene, increased slightly in transgenic Arabidopsis lines, while in WT it was enhanced and significantly higher than that in transformed plants from 3 dpi onward, see Figure 3C.

**Figure 4:**
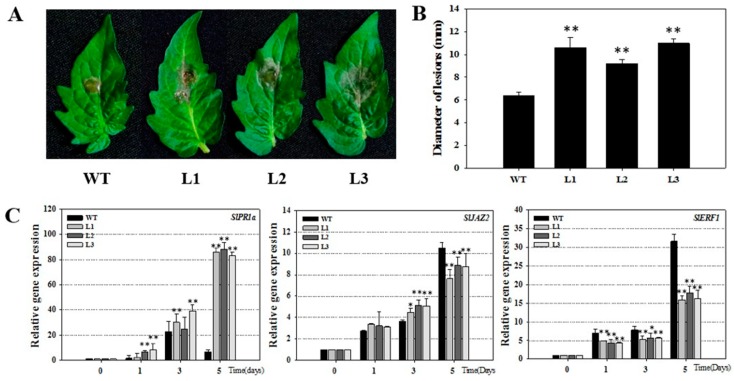
The assessment of *VqSTS36* transgenic tomato lines subjected to *Botrytis cinerea*. (**A**) The symptoms of the transformed tomato and an untransformed one subjected with *B. cinerea* after 4 days. (**B**) Statistics of lesions’ diameter. (**C**) Relative expression levels of defense-related genes in leaves collected at 0, 1, 3 and 5 dpi. Data represent mean values ± SD from three independent experiments. Asterisks indicate significant differences between WT and transgenic lines as determined by Student’s *t*-test (* 0.01 < *p* < 0.05, ** *p* < 0.01).

To verify the results for transgenic Arabidopsis, we detect the disease defense of tomato. We introduced *VqSTS36* gene into tomato (Micro-Tom, Supplementary Figure S1). *VqSTS36*-transformed tomato lines, along with wild-type plants were infected with *B. cinerea* and assessed for 5 days. After inoculation, leaf surfaces showed oil stains, and visible hypha, see Figure 4A. Furthermore, transgenic tomatoes displayed more serious disease symptoms than WT at 4 dpi, see Figure 4A,B. The diameter of lesion of WT was about 6.4 mm, which was smaller than transformed tomato lines (L1, L2, and L3 are about 10.6, 9.2, and 11 mm, respectively, see Figure 4B). To gain insight into the pathway controlling these alterations in *VqSTS36* transgenic tomato in response to *B. cinerea*, we detected the relative expression of several SA- and JA/ET-mediated genes at 0, 1, 3, and 5 dpi, respectively, see Figure 4C. Through qRT-PCR analysis, the relative expression level of *SlPR1a* [27] associated with SA-responsive gene in the transgenic tomato was significantly up-regulated from 1 dpi onward, and higher than those in wild-type tomato. Although, *SlJAZ2* and *SlERF1* [28] are maker genes regulated by JA/ET-mediated defense signaling pathways, were significantly up-regulated in both transformed and WT tomatoes following exposure to *B. cinerea*, the increase in transgenic lines was significantly smaller than WT at 5 dpi, see Figure 4C. The related gene expression suggested that *VqSTS36* may induce the plant’s immunity system through SA and JA signals at the early stage of the infection. However, with the corresponding enhancement of the SA responses, the JA pathway was suppressed. Therefore, the increases in *SlJAZ2* and *SlERF1*were slower than WT at 5 dpi, as shown in Figure 4C.

### 2.3. Over-Expression of VqSTS36 Enhanced the Tolerance of Arabidopsis Seedlings to Salt and Drought Stresses

**Figure 5:**
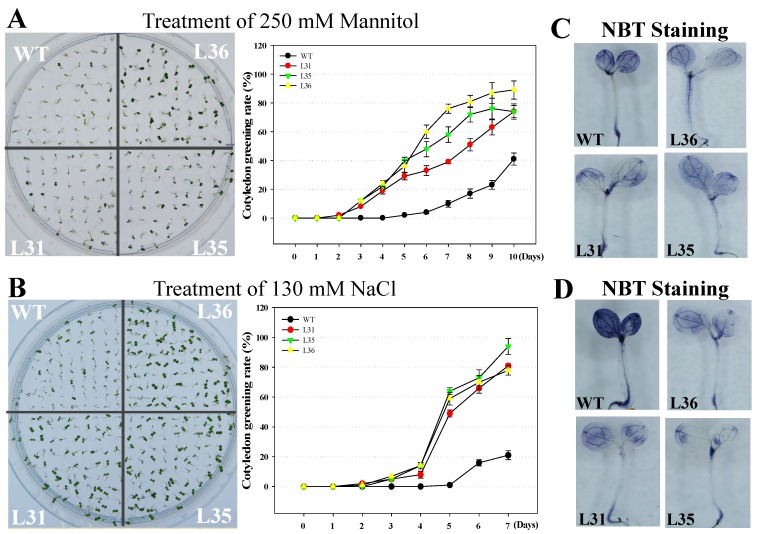
Cotyledon greening rates of *VqSTS36* transgenic Arabidopsis lines following the induction of osmotic stress. Homozygous seeds from *VqSTS36* transgenic lines (L31, L35, and L36) and untransformed controls (WT) were placed on MS medium added with 250 mM mannitol (**A**) and 130 mM NaCl (**B**), respectively, and assessed for cotyledon greening rates.Cotyledon greening rates were assessed daily following the initiation of each treatment. Representative images of plants were taken 10 days after seeds were plated on an osmotic stress-inducing medium. Data represent mean values from three independent experiments. (**C**) and (**D**) NBT staining for seedlings treated with drought and salt stresses.

The seed germination rate under osmotic stress is important for the tolerance of plants. So the T_3_ transgenic and WT Arabidopsis seeds were sown into the medium supplemented with 130 mM NaCl or 250 mM mannitol to simulate salts or arid environments, respectively. The results showed that the seed germination rate of transgenic Arabidopsis was significantly higher than that of WT, see Figure 5A,B. After 10 days from the time the seeds were subjected with Mannitol and NaCl stress, the accumulation of O_2_^−^ in transgenic lines was less than that in WT, see Figure 5C,D, indicating that the expression of *VqSTS36* in transgenic Arabidopsis was beneficial for increasing seed resistance to osmotic stress.

**Figure 6:**
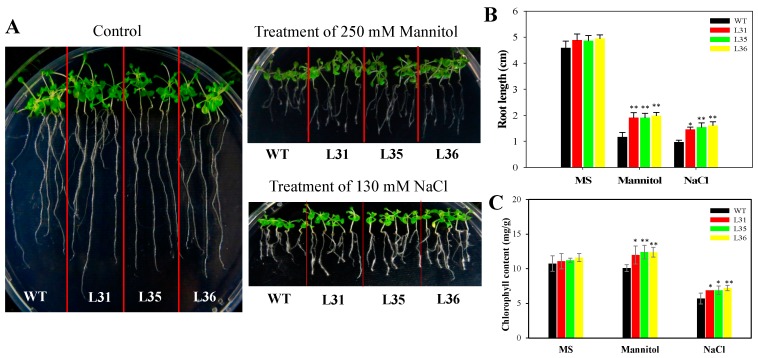
The effect of osmotic stress on seedlings of *VqSTS36* transgenic Arabidopsis seedlings. (**A**) Five-day old transgenic (L31, L35, and L36) and untransformed seedlings were transferred from MS plates to new MS plate, MS supplemented with 130 mM NaCl, 250 mM mannitol. Representative images were taken. (**B**) Root lengths were measured 7 days after the initiation of osmotic stress treatment. (**C**) 7 days old *VqSTS36* transgenic (L31, L35, and L36) and WT seedlings were transferred from the MS medium to an MS medium supplemented with 250 mM mannitol, 130 mM NaCl, and chlorophyll content was assessed 7 days following the initiation of osmotic stress treatments. Data represent mean values ± SD from three independent experiments. Asterisks indicate significant differences between WT and transgenic lines as determined by Student’s *t*-test (* 0.01 < *p* < 0.05, ** *p* < 0.01).

In this study, we also detected the changes of root length under osmotic stress to measure the resistance of transgenic *A. thaliana* seedlings. Drought and high concentrations of salt can inhibit plant root development and its nutrient absorption [13]. After 7 days of treatment with 130 mM NaCl and 250 mM mannitol, compared with control, the root length of transgenic and WT Arabidopsis seedlings were shortened, but the root length of *VqSTS36* transgenic seedlings were longer than that of WT, see Figure 6A,B. While chlorophyll content was determined to be significantly higher in *VqSTS36* transgenic lines than WT following osmotic stress, it was significantly reduced in transgenic lines, see Figure 6C. These results suggested that heterologous expression of VqSTS36 yields plants that are better able to withstand drought and salinity as evidenced by less chlorosis. The results showed that transgenic Arabidopsis increased its ability to resist osmotic stress.

### 2.4. Profile Expression of VqSTS36 Transgenic Arabidopsis Seedlings Respond to Osmotic Stress.

**Figure 7:**
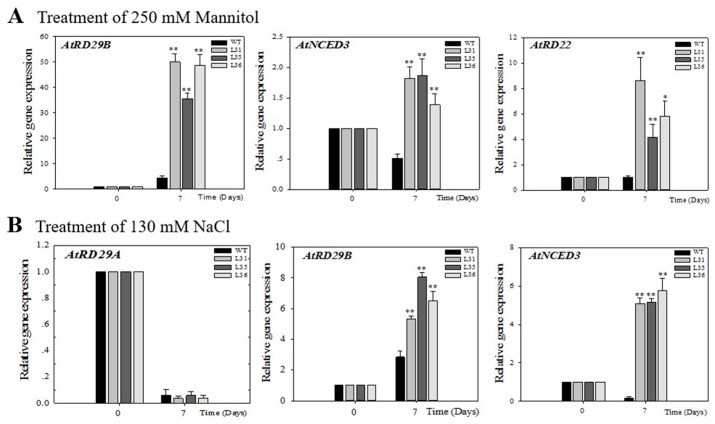
Assessment of the expression of stress-related genes in Arabidopsis seedlings after treatment with osmotic stress is shown in (**A**) and (**B**). The relative expression levels of osmotic stress-responsive genes were assayed in seedlings via qPCR 7 days following initiation of salt (**A**) and drought (**B**). Data represent mean values ± SD from three independent experiments. Asterisks indicate significant differences between WT and transgenic lines as determined by Student’s *t*-test (* 0.01 < *p* < 0.05, ** *p* < 0.01).

In order to explore the potential signal pathway of *VqSTS36* to enhance Arabidopsis stress tolerance resistance, this study analyzed the expression profile of stress-related genes, including *AtRD29A*, *AtRD29B*, *AtRD22*, and *AtNCED3* genes, in transgenic Arabidopsis seedlings after 7 days of salt and drought treatment, see Figure 7A,B. The *AtRD29A* and *AtRD29B* genes are important genes downstream of the ABA biosynthetic pathway [29], *AtRD22* is not only involved in the response of ABA signaling to abiotic stress but also has a response to dehydration [30]; *AtNCED3* is the indicator of ABA biosynthesis [31]. Real-time quantitative analysis showed that after drought treatment of Arabidopsis seedlings, the expression of *AtRD29B*, *AtNCED3*, and *AtRD22* in transgenic Arabidopsis were up-regulated by 35- to 50-fold, 1.4- to 1.9-fold and 4- to 8-fold, respectively, which was significantly higher than that of transcription level in WT seedlings, see Figure 7A. Under salt treatment, the transcription level of *AtRD29A* was down-regulated and showed no obvious difference between the transgenic and WT Arabidopsis seedlings. While the expression of *AtRD29B* was up-regulated 5- to 8-fold in *VqSTS36* over-expressing Arabidopsis seedlings, with a value higher than in WT. Besides, *AtNCED3* expression was increased 5-fold but was down-regulated in the WT, see Figure 7B.

## 3. Discussion

Pathogens disease, drought, and high salinity can cause serious damage to plant growth and development, fruit yield, and quality. Grapevine is an agriculturally economic fruit, which is easily attacked by biotic and abiotic stress from the environment. Fortunately, *STS* genes can not only enhance plant’s disease resistance [32] but also benefit human health by synthesizing phytoalexins [33]. Therefore, the research of functional *STS* genes can provide a genetic resource for new grape breeding in the future.

However, even if grapevines possess *STS* genes, different resistance are demonstrated by the varieties of species. For example, European varieties of grapevine are highly susceptible to powdery mildew, while 18 *Vitis* species derived from China were demonstrated carry resistance to powdery mildew [34]. In order to explore role of *STS* genes in disease resistance, *STS* genes have been successfully introduced in different species, such as white poplar [35], apple [36], lettuce [37], papaya [38], Arabidopsis [39], tomato [40,41], hop [42], grape [4], and kiwifruit [43]. Although, in most cases, the resistance of transgenic lines to fungal infections was increased to some extent, pathogen invasion was often not eradicated completely and instead morbidity were just partially alleviated or postponed [41,43]. *Vitis* stilbene synthase gene introduced into kiwifruit (*Actinidia deliciosa*) did not enhance the resistance to *B. cinerea* [43]. Heterologous expression of *STS* in transgenic tomatoes increased resistance against *Alternaria solani* but not *B. cinerea* [41]. Besides, after the inoculation of powdery mildew, the transcription level of *STS* genes was significantly enhanced in powdery mildew-susceptible *V. vinifera* compared to that in powdery mildew-resistant *V. aestivalis*, which implied that resistance to powdery mildew in *V. aestivalis* was not related to the changes in the transcriptome of the *STS* genes [44]. They also found that endogenous SA levels were higher in *V. aestivalis* than *V. vinifera* in the absence of powdery mildew, while SA levels were enhanced in *V. vinifera* post-infection of the fungi [44]. In our study, over-expressing *VqSTS36* in Arabidopsis showed an enhanced disease resistance to powdery mildew, see Figure 1B, but *VqSTS36* transgenic Arabidopsis and tomato lines instead increased the susceptibility to *Botrytis cinerea*, see Figure 2 and Figure 4A. It means that resistance to different pathogens of *VqSTS36* in transgenic plants may be associated with the different signal pathways in plants.

Both resistances to pathogens and susceptibility of *VqSTS36* were linked to the variation in the SA defense signal response in the transgenic plants. SA plays a pivotal role in plant defense as well as the activation of defense responses to biotrophic pathogens by inducing the expression of pathogenesis-related (PR) gene [45]. A flexible signaling network between SA and JA was demonstrated to allow plants to adjust their defense responses to invaders [46]. Biotrophic pathogens are more generally deterred by SA-mediated defenses, whereas necrotrophic pathogens are commonly sensitive to JA/ET-mediated responses [47,48]. Previous studies showed that both SA and JA messengers can be antagonistic. Induction of the SA response, either by pathogens inoculation or by application of exogenous SA, strongly restrained JA-related genes [49,50]. An exopolysaccharide, produced by *B. cinerea*, could induce the SA pathway response, which caused the promotion of disease morbidity. Plant cell death is believed to be facilitated by producing an oxidative burst activated by the host cells in reaction to *B.cinerea* [14]. In this study, both necrotrophic and biotrophic pathogens significantly induced the SA signaling pathway while inhibiting the JA response in *VqSTS36*-transformed Arabidopsis, which lead to an enhanced resistance against powdery mildew, but an increased susceptibility to *B. cinerea*, see Figure 1E, Figure 3C and Figure 4C. Identically, *VqSTS36* transgenic tomato also provided evidence.

UV irradiation can cause serious damage on the growth and development of plants by triggered reactive oxygen species (ROS), while it has been proven that *STS* gene and its production can protect plants by reducing the accumulation of ROS [51]. The antioxidant activity of *STS* gene in transgenic tomato was two times greater than that in untransformed ones [52]. Excess ROS activated by osmotic stress cause drought sensitivity [53,54], whereas low levels of ROS increases tolerance to osmotic stress [55]. Studies also proved that *STS* genes are linked with the improvement of resistance when plants are suffering from stress such as drought or high salinity. Osmotic stress lead to up-regulation of the expression of *STS* genes and activated stilbenoid biosynthesis [56,57,58]. In this current study, we found that *VqSTS36* significantly enhanced Arabidopsis seedling resistance to osmotic stress by the induction of ABA signaling response, see Figure 7, and reduced the ROS activated by osmotic stress, see Figure 5 and Figure 6. However, this tolerance did not show in mature transgenic Arabidopsis, and further research is still required to interpret the precise role of *STS* gene functioning in the tolerance to drought or salt stress.

## 4. Materials and Methods

### 4.1. Construction of Vector

Total RNA was extracted from *V. quinquangularis* cv. “Shang-24” leaf samples using the E.Z.N.A.^®^ Plant RNA Kit (Omega Biotech, Norcross, GA, USA). The first strand cDNA synthesis was carried out using PrimerScript^TM^RTase (TaKaRa Bio Inc., Dalian, China). Amplification of cDNA was conducted using 2 × *Taq* PCR MaterMix (BioSci Biotech, Hangzhou, China) and specific primers, see Supplementary Table S1.

The full-length grape *VqSTS36* coding sequence was amplified using gene-specific primers F1 (5′-CATGGCTTCAGTTGAGGAAATCAG-3′) and R1 (5′-GGGGGGATAATGAAACAGTGAGATA-3′) and 2×Taq PCR MasterMix (BioSci Biotech, Hangzhou, China). The PCR product was cloned into the pGEM^®^-T Easy vector (Promega, Madison, WI, USA). Amplified *VqSTS36* sequence using gene-specific primers with restriction sites F2 (5′-CGCGGATCCATGGCTTCAGTT GAGG-3′ *Sma* I site underlined) and R2 (5′-CTACCCGGGGTACCATTCCCCTTC AC-3′ *BamH* I site underlined). Following cloning, the *VqSTS36* sequence was inserted downstream of the CaMV 35S promoter in a pCambia 2300 vector (Cambia, Brisbane, Queensland, Australia; Figure 1A), then introduced into *Agrobacterium tumefaciens* (strain GV3101) using electroporation.

### 4.2. Arabidopsis Transformation

The above plant transformation vector was introduced into *A. thaliana* (Col-0) using the floral dip method [59]. We obtained 57 independent positive transgenic lines. To identify transgenic lines, T_1_ seeds were harvested and sown on MS medium added with 75 mg/L kanamycin. According to the PCR detection and phenotype of transgenic plants’ resistance against powdery mildew (*Golovinomyces. cichoracearum*), three T_3_ homozygous lines (L31, L35 and L36) were generated with universal representation and then utilized for subsequent experiments, wild-type (WT) plants were used as the untransformed control. All Arabidopsis plants were grown at 21 to 23 °C with a 16 h/8 h photoperiod (100 μmol m^−2^ s^−1^ photon flux density) at ~60% relative humidity (RH) on soil.

### 4.3. Transformed Arabidopsis Pathogens Inoculation

Four-week old transgenic *A. thaliana* and WT were infected with powdery mildew (*G. cichoracearum*) through gentle contact with leaves exhibiting disease symptoms. Subjected plants were incubated at 22 °C with ~80% RH for 3 days following inoculation, and then transferred to the proper condition with 30~40% RH. The response of *VqSTS36* transgenic plants and WT were monitored for 7 days. Each experiment was conducted in triplicate.

*B. cinerea* was cultured on potato dextrose agar medium at 25 °C in the dark. Conidial spore suspensions (2 × 10^7^ conidia/mL) were prepared with 14-day old cultures using sterile ddH_2_O as described previously [39]. Four-week old plants, including the three transgenic lines (L31, L35, L36) and WT were rinsed with 10 μL conidial suspension. Inoculated plants were incubated at 22 °C under a 16 h light and 8 h dark photoperiod and 80 to 90% RH. Disease incidence and lesion diameter were recorded daily. Each experiment was conducted in triplicate.

### 4.4. Detection of Cell Death and Superoxide Accumulation in Arabidopsis Leaves Treated with Pathogens

Following pathogen inoculation, using trypan blue and nitro blue tetrazolium (NBT) stainings to measure cell death and Superoxide anions (O_2_^−^) accumulation, respectively, in leaves detached from transgenic Arabidopsis and WT. Experiments involving powdery mildew and *B. cinerea* were carried out at 4 dpi and 3 dpi, respectively. In the case of trypan blue, staining was conducted as described previously [39]. Briefly, inoculated leaves were immersed in boiled trypan blue solution (a 1:1:1:1:1 ratio of water, trypan blue, phenol, glycerol, and lactic acid) for 2 to 3 min, and then were decolorized in 2.5 g/mL chloral hydrate for 1 to 2 days. For NBT staining, infected leaves were socked in HEPES buffer (pH 7.5) containing 6 mM NBT for 2 to 3 h [26]. Each experiment was conducted in triplicate.

### 4.5. Tomato Transformation

Tomato transformation was performed as described previously with some modification [60,61] (Ouyang et al. 2005; Zhang et al. 2006). Disinfecting tomato seeds two to three times with sterile water in a Petri dish, 30 sec with 70% ethanol, three times with sterilized water, and then 15 min with 10% sodium hypochlorite, before, finally, washing five times with sterile water. The sterilized seeds were grown on the 1/2MS medium (2.47 g/L 1/2MS powder, 10 g/L sucrose, 8 g/L agar, pH5.8) at 24 to 26 °C without light. When 50% of the seeds were germinated in a medium, they were grown at 24 to 26 °C with a 16/8 h photoperiod (1800 Lux) until the cotyledons were fully expanded.

The fully expanded cotyledons were extracted from 10-day old tomato, seedlings were cut with two incisions paralleled to the vein, and leaves were upturned and cultured on the pre-culture medium (4.433 g/L MS powder, 10 g/L sucrose, 8 g/L agar, 2 mg/L ZT, 0.2 mg/L IAA, 0.1 mM AS, pH 5.8) without light for 2 days. Tomato cotyledons were soaked in the transformed *A. tumefaciens* liquid (OD_600_ = 0.4–0.5) for 20 min, blotted dry on sterilized filter paper, subsequently grown on co-culture medium (4.433 g/L MS powder, 10 g/L sucrose, 8 g/L agar, 2 mg/L ZT, 0.2 mg/L IAA, 0.1 mM AS) for a further 2 days. Infected cotyledons were placed on screening medium (4.433 g/L MS powder, 10 g/L sucrose, 8 g/L agar, 2 mg/L ZT, 0.2 mg/L IAA, 200 mg/L cef + 50 mg/L Kana, pH5.8) for 10 days and sub-cultured every 3 weeks.

When resistance shoots reached 2 cm, cotyledons were excised from callus and transferred onto rooting medium (4.433 g/L MS powder, 10 g/L sucrose, 8 g/L agar, 0.2 mg/L IAA, 20 mg/L cef + 25 mg/L kanamycin, PH5.8). Plantlets with well-developed roots were finally transplanted to soil, harvested the seeds (T_1_), then sown on the medium added with 25 mg/L kanamycin to screen T_2_ plants.

### 4.6. Transformed Tomato Infected with Botrytis Cinerea

Using the same method as Arabidopsis plants infected with *B.* *cinerea* to prepare the conidial spore suspensions (2 × 10^7^ conidia/mL). Specifically, leaves separated from transgenic and wild-type tomato were rinsed with sterilized ddH_2_O, and then placed on a 1% agarose overlay in a Petri dish. A total of 10 L spore suspensions were inoculated on detached leaves, followed by culturing at 25 °C under a 16 h light and 8 h dark photoperiod, and RH kept at 90% to 95%. The disease incidence and lesion diameter were recorded daily and leaves were collected after 5 days for the analysis of transcription levels of related genes. Each experiment was conducted in triplicate.

### 4.7. Effect of Osmotic Stress on the Seeds and Seedlings of Transgenic Arabidopsis

To assess cotyledon greening rates of transformed Arabidopsis following salt and drought stress. T_3_ seeds of transgenic lines and WT with antiseptic to be soaked in 75% ethanol for 30 s, washed with sterilized ddH_2_O a total three times, then immersed in 10% NaClO for 3 to 5 min, and finally washed with sterilized ddH_2_O a total of five times. To detect the optimistic concentrations of mannitol and NaCl for cotyledon greening rates, both transgenic and WT seeds were sown on MS medium supplemented with different concentrations of mannitol and NaCl. Then, 120 sterilized seeds were sown on MS medium containing 250 mM mannitol and 130 mM NaCl to mimic salt and drought stress, respectively [26,39], and were incubated at 21 to 23 °C under a 16 h light and 8 h dark photoperiod. Cotyledon greening rates were recorded daily. Each experiment was conducted in triplicate.

To determine the response of seedlings to osmotic stress, four-day old transgenic and WT seedlings which had been cultivated on MS medium were transferred to either a new MS medium or an MS medium containing 250 mM mannitol or 130 mM NaCl, respectively. Root lengths were measured 6 days following both types of osmotic treatment. In each instance, experiments were carried out in triplicate.

For the measurement of the chlorophyll content of plants. *VqSTS36* transgenic lines and WT seeds were sown on MS medium plates, and 7-day old seedlings were subsequently transferred to flasks containing MS liquid medium supplemented with 130 mM NaCl or 250 mM mannitol. Seven days following the commencement of osmotic stress treatment, seedlings were collected for physiological assessments. To measure chlorophyll content, 0.1 g seedlings with their roots removed were submerged in 5 mL 96% ethanol and incubated at 4 °C until the seedlings turned white.

### 4.8. Analysis of Quantitative Real-Time PCR

Total RNA was extracted using the Ultrapure RNA kit (ComWin Biotech, Beijing, China), and first-strand cDNA synthesis was performed according to the TransScript (Transgene Biotech, Beijing, China), which contained 200 ng total RNA. Subsequent qPCR assays were carried out using 2 × TransStart Tip Green qPCR Supermix (Transgene Biotech). Gene-specific primers used are listed in the supplementary, Table S1. The PCR parameter is: 94 °C denaturing 30s; 94 °C, 5s; 60 °C, 30s, the cycle number is 40. Arabidopsis plants with *AtActin2* and tomato plants with *SlActin* as the internal reference genes, respectively.

### 4.9. Statistical Analysis

Data analysis and charts were made using the SigmaPlot software. Mean values (±SD) were determined from the data set for three replications. Differences between stress treatments were examined with Student’s *t*-test, and were considered statistically significant at *p* < 0.05.

## Supplementary Materials

The supplementary materials are available online at http://www.mdpi.com/1422-0067/19/10/2985/s1.

## References

[1] Sales J.M., Resurreccion A.V.A. (2014). Resveratrol in Peanuts. Crit. Rev. Food Sci..

[2] Kodan A., Kuroda H., Sakai F. (2002). A Stilbene Synthase from Japanese Red Pine (Pinus densiflora): Implications for Phytoalexin Accumulation and Down-regulation of Flavonoid Biosynthesis. Proc. Natl. Acad. Sci. USA.

[3] Sobolev V.S., Neff S.A., Gloer J.B. (2009). New Stilbenoids from Peanut (*Arachis hypogaea*) Seeds Challenged by An *Aspergillus caelatus* Strain. J. Agric. Food Chem..

[4] Xu W.R., Yu Y.H., Ding J.H., Hua Z.Y., Wang Y.J. (2010). Characterization of A Novel Stilbene Synthase Promoter Involved in Pathogen- and Stress-inducible Expression from Chinese Wild *Vitis pseudoreticulata*. Planta.

[5] Yu C.K., Springob K., Schmidt J., Nicholson R.L., Chu I.K., Yip W.K., Lo C. (2005). A Stilbene Synthase Gene (*SbSTS1*) Is Involved in Host and Nonhost Defense Responses in Sorghum. Plant Physiol..

[6] Corso M., Vannozzi A., Maza E., Vitulo N., Meggio F., Bouzayen M., Valle G., Bonghi C., Lucchin M. (2016). Transcriptome Pathways in Leaf and Root of Grapevine Genotypes with Contrasting Drought Tolerance. Acta Hortic..

[7] Ismail A., Riemann M., Nick P. (2012). The Jasmonate Pathway Mediates Salt Tolerance in Grapevines. J. Exp. Bot..

[8] Ji W., Wang Y.J. (2013). Breeding for Seedless Grapes Using Chinese Wild Vitis spp. II. In Vitro Embryo Rescue and Plant Development. J. Sci. Food Agric..

[9] Mullins P.R. (1992). The Effects of Advertising Restrictions on Tobacco Consumption. Br. J. Addict..

[10] Hann D.R., Gimenez-Ibanez S., Rathjen J.P. (2010). Bacterial Virulence Effectors and Their Activities. Curr. Opin. Plant Biol..

[11] Bouarab K., Melton R., Peart J., Baulcombe D., Osbourn A. (2002). A Saponin-detoxifying Enzyme Mediates Suppression of Plant Defences. Nature.

[12] Grant M., Lamb C. (2006). Systemic Immunity. Curr. Opin. Plant Biol..

[13] D’Onofrio C., Cox A., Davies C., Boss P.K. (2009). Induction of Secondary Metabolism in Grape Cell Cultures by Jasmonates. Funct. Plant Biol..

[14] El Oirdi M., El Rahman T.A., Rigano L., El Hadrami A., Rodriguez M., Daayf F., Bouarab K. (2011). *Botrytis Cinerea* Manipulates The Antagonistic Effects between Immune Pathways to Promote Disease Development in Tomato. Plant Cell.

[15] Giraud E., Ivanova A., Gordon C.S., Whelan J., Considine M.J. (2012). Sulphur Dioxide Evokes A Large Scale Reprogramming of The Grape Berry Transcriptome Associated with Oxidative Signalling and Biotic Defence Responses. Plant Cell Environ..

[16] Kiselev K.V., Tyunin A.P., Karetin Y.A. (2015). Salicylic Acid Induces Alterations in The Methylation Pattern of The *VaSTS1*, *VaSTS2*, and *VaSTS10* Genes in *Vitis amurensis* Rupr. Cell Cultures. Plant Cell Rep..

[17] Chaves M.M., Zarrouk O., Francisco R., Costa J.M., Santos T., Regalado A.P., Rodrigues M.L., Lopes C.M. (2010). Grapevine under Deficit Irrigation: Hints from Physiological and Molecular Data. Ann. Bot..

[18] Walker R.R., Blackmore D.H., Clingeleffer P.R., Correll R.L. (2002). Rootstock Effects on Salt Tolerance of Irrigated Field-grown Grapevines (*Vitis Vinifera* L.cv. Sultana).: 1.Yield and Vigour Inter-Relationships. Aust. J. Grape Wine Res..

[19] Kumar M., Choi J., An G., Kim SR. (2017). Ectopic Expression of *OsSta2* Enhances Salt Stress Tolerance in Rice. Front. Plant Sci..

[20] Xie R., Zhang J., Ma Y., Pan X., Dong C., Pang S., He S. (2017). Combined Analysis of mRNA and miRNA Identifies Dehydration and Salinity Responsive Key Molecular Players in Citrus Roots. Sci. Rep..

[21] Kumar M. (2013). Crop Plants and Abiotic Stresses. J. Biomol. Res. Ther..

[22] Corso M., Vannozzi A., Maza E., Vitulo N., Meggio F., Pitacco A., Telatin A., D’ Angelo M., Feltrin E., Negri A.S. (2015). Comprehensive Transcript Profiling of Two Grapevine Rootstock Genotypes Contrasting in Drought Susceptibility Links the Phenylpropanoid Pathway to Enhanced Tolerance. J. Exp. Bot..

[23] Blum A., Belhassen E. (1996). Drought Tolerance in Higher Plants: Genetical, Physiological and Molecular Biological Analysis. Crop Responses to Drought and the Interpretation of Adaptation.

[24] Cramer G.R., Ergül A., Grimplet J., Tillett R.L., Tattersall E.A.R., Bohlman M.C., Vincent D., Sonderegger J., Evans J., Osborne C. (2007). Water and Salinity Stress in Grapevines: Early and Late Changes in Transcript and Metabolite Profiles. Funct. Integr. Genom..

[25] Tillett R., Ergul A., Albion R., Schlauch K., Cramer G., Cushman J. (2011). Identification of Tissue-Specific, Abiotic Stress-Responsive Gene Expression Patterns in Wine Grape. *Vitis vinifera* L. Based on Curation and Mining of Large-Scale EST Data Sets. BMC Plant Biol..

[26] Huang L., Zhang S.L., Singer S.D., Yin X.J., Yang J.H., Wang Y.J., Wang X.P. (2016). Expression of The Grape *VqSTS21* Gene in Arabidopsis Confers Resistance to Osmotic Stress and Biotrophic Pathogenes but Not *Botrytis Cinerea*. Front. Plant Sci..

[27] Gonorazky G., María C.G., Abd-El-Haliem A.M., Joosten M.H.A.J., Laxalt A.M. (2016). Silencing of The Tomato Phosphatidylinositol-Phospholipase C2 (SlPLC2) Reduces Plant Susceptibility to *Botrytis Cinerea*. Mol. Plant Pathol..

[28] Zhang H., Hong Y., Huang L., Liu S., Li X., Ouyang Z., Song F., Zhang Y., Li D. (2015). Tomato Histone *H2B* Monoubiquitination Enzymes *SlHUB1* and *SlHUB2* Contribute to Disease Resistance Against *Botrytis Cinerea* through Modulating The Balance between SA- and JA/ET-Mediated Signaling Pathways. BMC Plant Biol..

[29] Stark L.R., Brinda J.C., McLetchie D.N., Oliver M.J. (2012). Extended Periods of Hydration Do not Elicit Dehardening to Desiccation Tolerance in Regeneration Trials of The Moss *Syntrichia Caninervis*. Int. J. Plant Sci..

[30] Harshavardhan VT., Son LV., Seiler C., Junker A., Weigelt-Fischer K., Klukas C., Altmann T., Sreenivasulu N., Bäumlein H., Kuhlmann M. (2014). AtRD22 and AtUSPL1, Members of The Plant-Specific BURP Domain Family Involved in *Arabidopsis Thaliana* Drought Tolerance. PLoS ONE.

[31] Endo A., Sawada Y., Takahashi H., Okamoto M., Ikegami K., Koiwai H., Nambara E. (2008). Drought Induction of Arabidopsis 9-*Cis*-Epoxycarotenoid Dioxygenase Occurs in Vascular Parenchyma Cells. Plant Physiol..

[32] Jeandet P., Douillt-Breuil A.C., Bessis R., Debord S., Sbaghi M., Adrian M. (2002). Phytoalexins from The *Vitaceae*: Biosynthesis, Phytoalexin Gene Expression in Transgenic Plants, Antifungal Activity, and Metabolism. J. Agric. Food Chem..

[33] Shankar S., Singh G., Srivastava R.K. (2007). Chemoprevention by Resveratrol: Molecular Mechanisms and Therapeutic Potential. Front. Biosci..

[34] Wang Y.J., He P.C. (1997). Study on Inheritance of leaves’ resistance to powdery mildew in Chinese native wild Vitis species. Agric. Sci. China.

[35] Giorcelli A., Sparvoli F., Mattivi F., Tava A.B.A., Vrhovsek U., Calligari P., Bollini R., Confalonieri M. (2004). Expression of the Stilbene Synthase (*StSy*) Gene from Grapevine in Transgenic White Poplar Results in High Accumulation of the Antioxidant Resveratrol Glucosides. Transgenic Res..

[36] Briviba K., Fleschhut J., Schönherr J., Jacobsen H., Kiesecker H., Szankowski I. (2003). Transformation of Apple (*Malus domestica* Borkh.) with the Stilbene Synthase Gene from Grapevine (*Vitis vinifera* L.) and A PGIP Gene from Kiwi (*Actinidia deliciosa*). Plant Cell Rep..

[37] Liu S., Hu Y., Wang X., Zhong J., Lin Z. (2006). High Content of Resveratrol in Lettuce Transformed with A Stilbene Synthase Gene of Parthenocissus Henryana. J. Agric. Food Chem..

[38] Zhu Y.J., Agbayani R., Jackson M.C., Tang C.S., Moore P.H. (2004). Expression of the Grapevine Stilbene Synthase Gene *VST1* in Papaya Provides Increased Resistance against Diseases Caused by *Phytophthora Palmivora*. Planta.

[39] Guo R.R., Tu M.X., Wang X.H., Zhao J., Wan R., Li Z., Wang Y.J., Wang X.P. (2016). Ectopic Expression of A Grape Aspartic Protease Gene, AP13, in *Arabidopsis thaliana* Improves Resistance to Powdery Mildew but Increases Susceptibility to *Botrytis cinerea*. Plant Sci..

[40] Ingrosso I., Bonsegna S., De Domenico S., Laddomada B., Blando F., Santino A., Giovinazzo G. (2011). Over-expression of A Grape Stilbene Synthase Gene in Tomato Induces Parthenocarpy and Causes Abnormal Pollen Development. Plant Physiol. Biochem..

[41] Thomzik J.E., Stenzel K., Stoecker R., Schreier P.H., Hain R., Stahl D.J. (1997). Synthesis of Grapevine Phytoalexin in Transgenic Tomatoes (Lycopersicon esculentum Mill.) Conditions Resistance against Phytophthora Infestans. Physiol. Mol. Plant Path..

[42] Schwekendiek A., Spring O., Heyerick A., Pickel B., Pitsch N., Peschke F., De Keukeleire D., Weber G. (2007). Constitutive Expression of A Grapevine Stilbene Synthase Gene in Transgenic Hop (*Humulus lupulus* L.) Yields Resveratrol and Its Derivatives in Substantial Quantities. J. Agric. Food Chem..

[43] Kobayashi S., Ding C.K., Nakamura Y., Nakajima I., Matsumoto R. (2000). Kiwifruits (*Actinidia deliciosa*) Transformed with A *Vitis* Stilbene Synthase Gene Produce Piceid (*resveratrol-glucoside*). Plant Cell Rep..

[44] Fung R.W.M., Gonzalo M., Fekete C., Kovacs L.G., He Y., Marsh E., Mclntyre L.M., Schachtman D.P., Qiu W. (2008). Powdery Mildew Induces Defense-Oriented Reprogramming of the Transcriptome in a Susceptible but Not in a Resistant Grapevine. Plant Physiol..

[45] Vicente M.R.S., Plasencia J. (2011). Salicylic Acid beyond Defence: Its Role in Plant Growth and Development. J. Exp. Bot..

[46] Bari R., Jones J. (2009). Role of Plant Hormones in Plant Defence Responses. Plant Mol. Biol..

[47] Glazebrook J. (2005). Contrasting Mechanisms of Defense against Biotrophic and Necrotrophic Pathogens. Annuel Rev. Phytopath..

[48] Koornneef A., Leon-Reyes A., Ritsema T., Verhage A., Otter F.C.D., Van Loon L.C., Pieterse C.M.J. (2008). Kinetics of Salicylate-Mediated Suppression of Jasmonate Signaling Reveal a Role for Redox Modulation. Plant Physiol..

[49] Koornneef A.,  Pieterse C.M. (2008). Cross Talk in Defense Signaling. Plant Physiol..

[50] Zarate S., Kempema L.A., Walling L.L. (2007). Silverleaf Whitefly Induces Salicylic Acid Defenses and Suppresses Effectual Jasmonic Acid Defenses. Plant Physiol..

[51] Chang X.L., Heene E., Qiao F., Nick P. (2011). The Phytoalexin Resveratrol Regulates the Initiation of Hypersensitive Cell Death in Vitis Cell. PLoS ONE.

[52] Morelli R., Das S., Bertelli A., Bollini R., Lo Scalzo R., Das D.K., Falchi M. (2006). The Introduction of The Stilbene Synthase Gene Enhances the Natural Antiradical Activity of *Lycopersicon Esculentum* Mill. Mol. Cell. Biochem..

[53] Sharma P., Jha A.B., Dubey R.S., Pessarakli M. (2012). Reactive Oxygen Species, Oxidative Damage, and Antioxidative Defense Mechanism in Plants under Stressful Conditions. J. Bot..

[54] You J., Zong W., Hu H.H., Li X.H., Xiao J.H., Xiong L.Z. (2014). A STRESS-RESPONSIVE NAC1-Regulated Protein Phosphatase Gene Rice Protein Phosphatase18 Modulates Drought and Oxidative Stress Tolerance through Abscisic Acid-Independent Reactive Oxygen Species Scavenging in Rice. Plant Physiol..

[55] Neill S.J., Desikan R., Clarke A., Hurst R.D., Hancock J.T. (2002). Hydrogen Peroxide and Nitric Oxide as Signalling Molecules in Plants. J. Exp. Bot..

[56] Deis L., Cavagnaro B., Bottini R., Wuilloud R., Silva M.F. (2011). Water Deficit and Exogenous ABA Significantly Affect Grape and Wine Phenolic Composition under in Field and In-vitro Conditions. Plant Growth Regul..

[57] Dubrovina A.S., Kiselev K.V., Khristenko S.V. (2013). Expression of Calcium-Dependent Protein Kinase (CDPK) Genes under Abiotic Stress Conditions in Wild-Growing Grapevine Vitis Amurensis. J. Plant Physiol..

[58] Hatmi S., Trotel-Aziz P., Villaume S., Couderchet M., Clement C., Aziz A. (2014). Osmotic Stress-Induced Polyamine Oxidation Mediates Defence Responses and Reduces Stress-Enhanced Grapevine Susceptibility to *Botrytis Cinerea*. J. Exp. Bot..

[59] Clough S.J., Bent A.F. (1998). Floral Dip: A Simplified Method for Agrobacterium-Mediated Transformation of Arabidopsis Thaliana. Plant J..

[60] Ouyang B., Chen Y.H., Li H.X., Qian C.J., Huang S.L., Ye Z.B. (2005). Transformation of Tomatoes with Osmotin and Chitinase Genes and Their Resistance to Fusarium Wilt. J. Hortic. Sci. Biotech..

[61] Zhang Y., Li H., Ouyang B., Lu Y., Ye Z. (2006). Chemical-Induced Autoexcision of Selectable Markers in Elite Tomato Plants Transformed with a Gene Conferring Resistance to Lepidopteran Insects. Biotechnol. Lett..

