# Supplementary material for: Expression of a Grape VqSTS36-Increased Resistance to Powdery Mildew and Osmotic Stress in Arabidopsis but Enhanced Susceptibility to Botrytis cinerea in Arabidopsis and Tomato
Source: Int J Mol Sci. 2018 Sep 30;19(10):2985. doi: 10.3390/ijms19102985 (PMC6213015; doi:10.3390/ijms19102985)
Supplement: Supplementary file 1 [file ijms-19-02985-s001.pdf]

Supplementary Materials

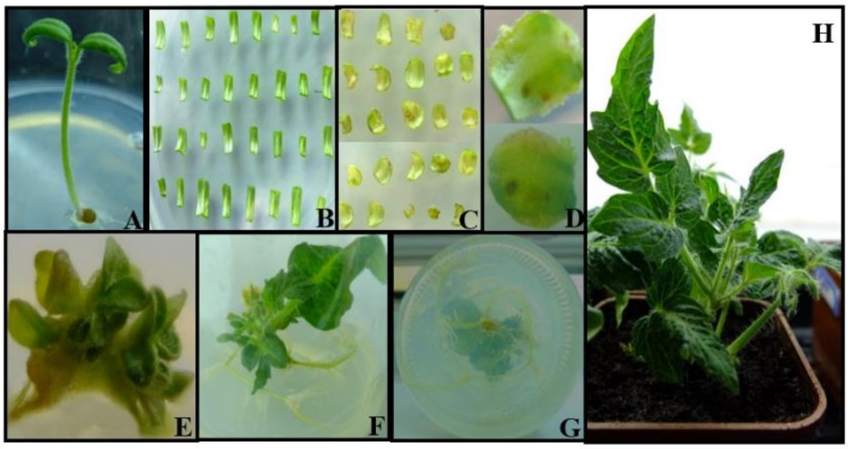

Supplementary Figure 1. The process for forming *VqSTS36* transgenic tomato. (**A**) 10-days WT tomato seedlings. (**B**)Cotyledons were cut for preculturing. (**C**) Cotyledons were transferred and grown on MS medium added with Kana. (**D**) Callus growth on selection medium. (**E**) The generation of resistant shoots. (**F–G**) Resistant shoots were transferred on medium for rooting, and resistant plant rooting. (**H**)Plants that grow well were transplanted into the soil.

Table S1. Primers used for RT-PCR.

Genes	Forwards primers（5′-3′）	Reverse primers（5′-3′）
AtPR1	AACTACGCTGCGAACACGTG	TCACTTTGGCACATCCGAGTC
AtNPR1	CTTCCGTGACCTTGATCCTTTCT	CAGCGATCTTGCCATTAGGATC
AtPDF1.2	GAAGCACAGAAGTTGTGCGA	TGTAACAACAACGGGAAAATAAACA
AtRD22	GGTTCGGAAGAAGCGGAGAT	AGTGGAAACAGCCCTGACGT
AtRD29A	AAGCAATGAGCATGAGCAAG	GGAAGACACGACAGGAAACAC
AtRD29B	ACGACGGAAACATCGGACT	CTTCACCACCAGGAGCAAA
AtNCED3	TTGATGCTCCAGATTGCTTC	GTCCACAGAAAAGCATAGCAG
AtActin	AGTGTCTGGATCGGTGGTTC	CCCCAGCTTTTTAAGCCTTT
SlPR1a	TCTTGTGAGGCCCAAAATTC	ATAGTCTGGCCTCTCGGACA
SlICS1	TCGCCGGCATTCATTGGAAACA	GCACTCCCGTACTATAGCAAAC
SlJAZ2	GGAAACCTGATCAACCAGAG	GGGGTTCTGTTTGTTGGCTA
SlERF1	TGGAGTTAGAAAGAGGCCATGG	CCCTCATTGATAATGCGGCTTG
SlActin	GCAGGACGTGACCTCA	GGAAGGTGCTGAGGGAA
